# Comparison of two strategies for managing in-hospital cardiac arrest

**DOI:** 10.1038/s41598-021-02027-2

**Published:** 2021-11-18

**Authors:** Jafer Haschemi, Ralf Erkens, Robert Orzech, Jean Marc Haurand, Christian Jung, Malte Kelm, Ralf Westenfeld, Patrick Horn

**Affiliations:** 1 Department of Cardiology, Pulmonology and Vascular Medicine, Medical Faculty of the Heinrich Heine University, Moorenstr. 5, 40225 Duesseldorf, Germany; 2 Cardiovascular Research Institute, Medical Faculty of the Heinrich-Heine University, Düsseldorf, Germany

**Keywords:** Health care, Outcomes research

## Abstract

In-hospital cardiac arrest (IHCA) is associated with poor outcomes. There are currently no standards for cardiac arrest teams in terms of member composition and task allocation. Here we aimed to compare two different cardiac arrest team concepts to cover IHCA management in terms of survival and neurological outcomes. This prospective study enrolled 412 patients with IHCA from general medical wards. From May 2014 to April 2016, 228 patients were directly transferred to the intensive care unit (ICU) for ongoing resuscitation. In the ICU, resuscitation was extended to advanced cardiac life support (ACLS) (Load-and-Go [LaG] group). By May 2016, a dedicated cardiac arrest team provided by the ICU provided ACLS in the ward. After return of spontaneous circulation (ROSC), the patients (n = 184) were transferred to the ICU (Stay-and-Treat [SaT] group). Overall, baseline characteristics, aetiologies, and characteristics of cardiac arrest were similar between groups. The time to endotracheal intubation was longer in the LaG group than in the SaT group (6 [5, 8] min versus 4 [2, 5] min, p = 0.001). In the LaG group, 96% of the patients were transferred to the ICU regardless of ROSC achievement. In the SaT group, 83% of patients were transferred to the ICU (p = 0.001). Survival to discharge did not differ between the LaG (33%) and the SaT (35%) groups (p = 0.758). Ultimately, 22% of patients in the LaG group versus 21% in the SaT group were discharged with good neurological outcomes (p = 0.857). In conclusion, we demonstrated that the cardiac arrest team concepts for the management of IHCA did not differ in terms of survival and neurological outcomes. However, a dedicated (intensive care) cardiac arrest team could take some load off the ICU.

## Introduction

In-hospital cardiac arrest (IHCA) is a major adverse event with an incidence of 1–6/1000 hospital admissions^1^. Mortality after IHCA is high, and the neurological outcome after the return of spontaneous circulation (ROSC) remains dismal^2,3^. Approximately every fourth IHCA patient survives to discharge^4,5^. Strategies to manage out-of-hospital cardiac arrest (OHCA) have been widely researched^6,7^, but little is known about the best management of IHCA to improve outcomes. Outcomes might be affected by patient characteristics^3,8–10^, cardiac arrest timepoint and location^11^, and cardiac arrest team performance. Ensuring adequate basic life support (BLS) and subsequently employing advanced cardiac life support (ACLS) according to the current resuscitation guidelines are of critical importance^12,13^.

Improving cardiac arrest team performance may increase survival. However, team compositions vary among hospitals and countries^14^. Currently, there are no international standards for IHCA teams in member composition and task allocation. It remains unclear how hospitals should assemble cardiac arrest teams to guarantee the optimal management of patients suffering from IHCA. Should IHCA management be organised based on OHCA strategies with a dedicated team that comes to the patient and continues ACLS on-site (Stay-and-Treat [SaT])? Alternatively, might it be advantageous when the patient is transferred as soon as possible (with ongoing cardiopulmonary resuscitation [CPR]) to an intensive care unit (ICU) where CPR can be continued and extended with higher levels of staff and equipment resources (Load-and-Go [LaG])? The latter concept could mean poorer chest compressions during transportation, but that effect might be compensated for by higher competency at ICU arrival and a higher possibility of treating reversible causes in the ICU.

This study aimed to compare the two different IHCA team concepts, CPR survival and neurological outcomes, at a single university hospital.

## Methods

In this before-and-after study, we prospectively enrolled all comers with IHCA between May 2014 and April 2018 in an internal medicine or neurological general ward at the university hospital. The patients were grouped according to cardiac arrest team concept. Patient characteristics, baseline data, and event data were assessed using medical records and event protocols. This study was conducted in accordance with the guidelines of the Declaration of Helsinki and received approval from the University of Düsseldorf Committee on Human Research (study number 2018-112-RetroDEuA), which waived the requirement for informed patient consent due to our use of anonymised data collected during critical care hospitalisation.

### Cardiac arrest team concepts (LaG versus SaT)

**Figure 1:**
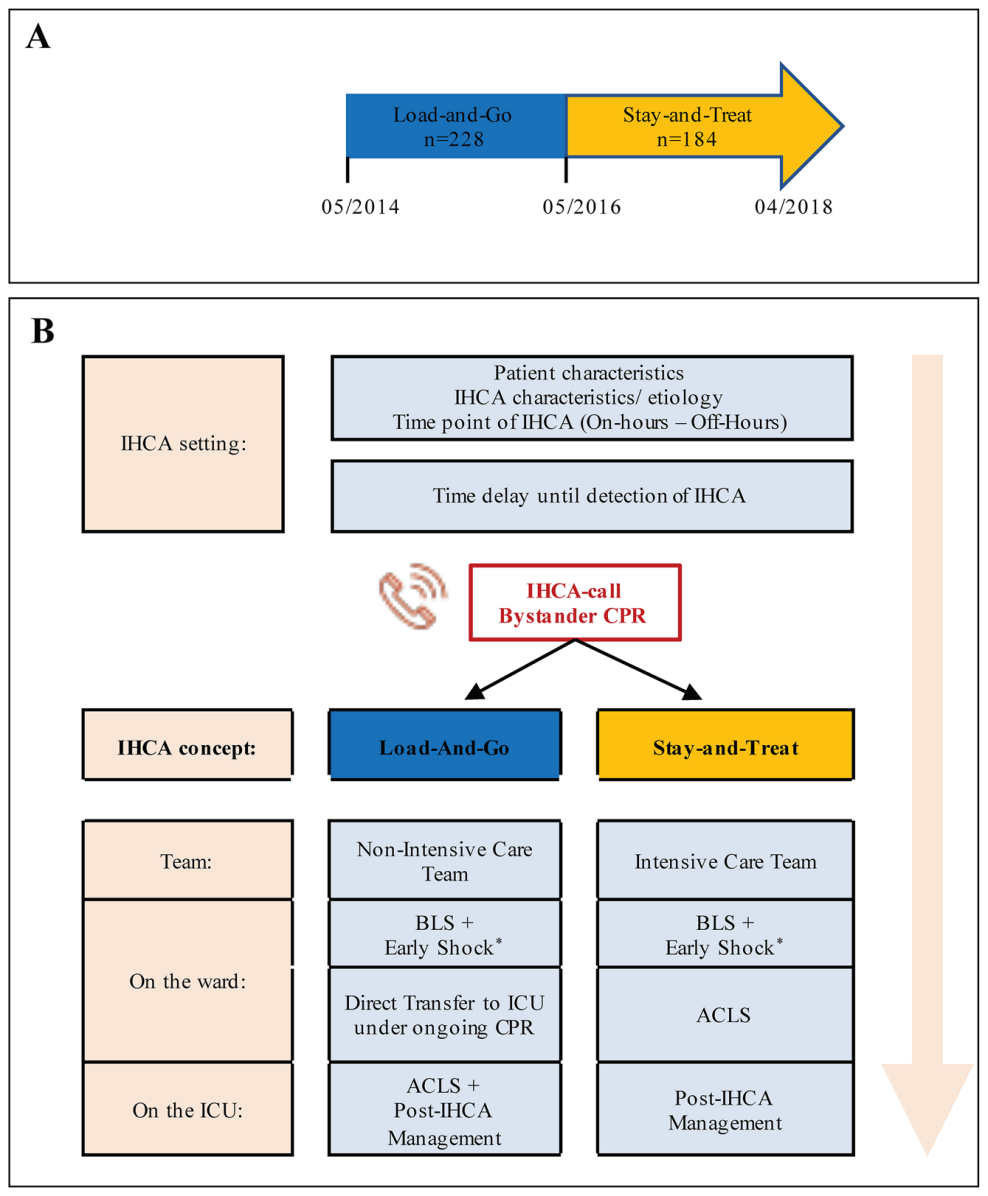
Cardiac arrest team concepts for the management of IHCA. (**A**) From May 2014 to April 2016, IHCA patients were supplied by a Load-and-Go (LaG) concept. From May 2016 to April 2018, the ICU provided a cardiac arrest team that managed IHCA using a Stay-and-Treat (SaT) concept. (**B**) Outcome of IHCA was determined by the setting at which IHCA occurred, time delay until detection and IHCA call was performed, and possibly by the characteristics of the IHCA team concept (focus of the present study). *IHCA* In-hospital cardiac arrest, *CPR* cardiopulmonary resuscitation, *ICU* Intensive Care unit, *ACLS* Advanced cardiac life support.

From May 2014 to April 2016, IHCA patients were treated by a cardiac arrest team with various staff compositions (LaG group) (Fig. 1), while the rapid initiation of CPR and early defibrillation were performed by nurses and physicians in the wards (non-intensive care specialists from the internal medicine or neurology department). During nights and weekends, the nurses, house officers on duty, and senior house officers on duty (from the internal medicine department) performed BLS and early defibrillation. Staff members are trained once a year for BLS. The patients were directly transferred to the ICU (after the achievement of early return of spontaneous circulation [ROSC] or with ongoing manual CPR. The airway was secured with a laryngeal mask or a single bag mask valve ventilation. In the ICU, the resuscitation was continued and extended to ACLS.

From May 2016 to April 2018, the ICU was responsible for responding to all cardiac arrests on the wards, and provided a dedicated cardiac arrest team consisting of an experienced physician and nurses from the ICU with fixed task assignments (Fig. 1) (SaT group): The team supported and extended CPR to ACLS on-site. CPR measures were continued or terminated according to the cardiac arrest team’s assessment, and cases of death were recorded. After ROSC achievement, the patient was transferred to the ICU.

### Primary and secondary endpoints

The primary endpoint of this study was survival to discharge. The secondary endpoints were the proportion of patients who achieved ROSC, time to achieve ROSC, and neurological outcome. Neurological outcomes were assessed according to peak neuron-specific enolase (NSE) levels and the cerebral performance category (CPC) scale. The CPC scale was determined by chart review of the neurological assessment performed by a physician at the time of discharge. In cases of missing chart reviews, a structured interview was conducted. The five-point CPC scale assesses brain recovery by assessing the functional and cognitive aspects of brain function^15^. CPC 1 was defined as good cerebral performance (conscious, alert, able to work, mild neurologic or psychologic deficit); CPC 2, moderate cerebral disability (conscious, sufficient cerebral function for independent activities of daily life, able to work in a sheltered environment; CPC 3, severe cerebral disability (conscious, dependent on others for daily support because of impaired brain function/progression from ambulatory state to severe dementia or paralysis; CPC 4, coma or vegetative state (any degree of coma without the presence of all brain death criteria, unawareness, vegetative state without interaction with environment, spontaneous eye opening and sleep/awake cycles; CPC 5, brain death (apnoea, areflexia, electroencephalography silence). A poor neurological outcome was defined as a CPC ≥ 3 at discharge.

### Statistics

Categorical variables are reported as absolute values and percentages, whereas continuous data are expressed as median (interquartile range). Categorical data were compared using the χ^2^ test or Fisher’s exact test. The D’Agostino & Pearson omnibus normality test was used to assess the distribution of the continuous variables. In cases of normal distribution, Student’s unpaired t-test was performed to compare the means between the two groups. Continuous variables that did not follow a normal distribution were compared using the Mann–Whitney U test. Cox regression analysis was used to identify whether variables associated with hospital mortality after IHCA differed between the LaG and SaT groups. Variables with a p-value < 0.1 in the univariate analysis and variables known or thought to be associated with mortality after IHCA were included in the multivariable model. Statistical significance was set at P < 0.05. The statistical analyses were performed using SPSS® Statistics 25 (IBM, Armonk, NY, USA) and Prism® (GraphPad, San Diego, CA, USA).

## Results

### Patients’ characteristics

From May 2014 to April 2016, 24,590 patients were admitted to the internal medicine or neurology departments of the university hospital. During this period, 228 patients had IHCA (LaG group). Between May 2016 and April 2018, 25,251 patients were admitted, and 184 cases of IHCAs occurred (SaT group). This means that the occurrence of IHCA decreased from 0.9 per 1000 patient admissions in the period of the LaG concept to 0.7 per 1000 patients (p = 0.014) in the period of the SaT concept.

Overall, the baseline characteristics were similar between the two groups (Table 1). The median age was 75 (64, 81) years in the LaG group versus 75 (64, 82) years in the SaT group (p = 0.730). The LaG and SaT groups included 62% and 64% male patients, respectively (p = 0.716). The aetiology of cardiac arrest did not differ between groups: IHCA had a cardiac aetiology in 120 patients (53%) versus 82 patients (46%), respectively (p = 0.103). The primary rhythm was shockable in 69 patients (30%) in the LaG group versus 63 patients (34%) in the SaT group (p = 0.390).

**Table 1:** Baseline characteristics of the study population.

Patient characteristics	Complete cohort n = 412	LaG-group n = 228	SaT-group n = 184	p-value
Age (years)	75 (64, 81)	75 (64, 81)	75 (64, 82)	0.471
Women/ male, n/n (%/%)	154/ 258 (37/63)	87/141 (38/62)	67/ 117 (36/64)	0.716
CAD, n (%)	242 (59)	140 (61)	102 (55)	0.221
PAD, n (%)	54 (13)	32 (14)	22 (12)	0.534
Arterial hypertension, n (%)	334 (81)	190 (83)	144 (78)	0.191
Diabetes mellitus, n (%)	87 (21)	50 (22)	37 (20)	0.652
End-stage renal failure, n (%)	31 (8)	18 (8)	13 (7)	0.751
GFR (ml/min)	45 (32, 67)	45 (26, 66)	43 (22, 76)	0.197
Haemoglobin (g/dl)	10.7 (9.0, 12.0)	10.6 (8.4, 12.3)	11.0 (9.2, 12.0)	0.143
C-reactive protein (mg/dl)	5.4 (1.7, 10.7)	5.1 (1.1, 9.7)	5.9 (2.4, 11.1)	0.889
Troponin (ng/l)	124 (53, 325)	122 (52, 299)	167 (52, 389)	0.414

Categorical variables are reported as absolute values and percentages, whereas continuous data are expressed as median with interquartile range.

*IHCA* In-hospital cardiac arrest, *LaG* Load-and-Go, *SaT* Stay-and-Treat, *CAD* coronary artery disease, *PAD* peripheral arterial disease, *GFR* glomerular fraction rate.

### Main results

The time from team arrival to first shock (if indicated) and the first administration of epinephrine did not differ between groups: 1 (0.5, 1) min versus 1 (0.5, 1) min, (p = 0.422), and 3 (2, 5) min versus 3 (3, 4) min (p = 0.143) (Table 2). The time to endotracheal intubation was longer in the LaG group than in the SaT group (6 [5, 8] min versus 4 [2, 5] min, p = 0.001).

**Table 2:** IHCA characteristics.

IHCA characteristics	Complete cohort n = 412	LaG-group n = 228	SaT-group n = 184	p-value
Cardiac arrest etiology, n (%)	202 (49)	120 (53)	82 (46)	0.103
Primary shockable rhythm, n (%)	132 (32)	69 (30)	63 (34)	0.390
Time to first shock (min)	1.0 (0.5, 1.0)	1.0 (0.5, 1.0)	1.0 (0.5, 1.0)	0.422
Time to first epinephrine administration (min)	3 (2, 4)	3 (2, 5)	3 (3, 4)	0.143
Time to endotracheal intubation (min)	5 (3, 7)	6 (5, 8)	4 (2, 5)	*0.001
Patients with ROSC, n (%)	311 (75)	168 (74)	143 (78)	0.344
Time to ROSC (min)	10 (3, 29)	15 (5, 30)	10 (2, 20)	0.114
Phosphat after ROSC (mmol/l)	1.9 (1.1, 2.5)	1.8 (1.2, 2.5)	2.0 (1.0, 2.4)	0.921
Lactate after ROSC (mg/l)	8.7 (3.6, 12.7)	9.4 (4.5, 12.5)	6.7 (2.7, 13.0)	0.281
Transfer to ICU n, (%)	373 (91)	220 (96)	153 (83)	*0.001

Categorical variables are reported as absolute values and percentages, whereas continuous data are expressed as median with interquartile range. * indicates p ≤ 0.05 between LaG group and SaT group. Significant values are in bold.

*IHCA* In-hospital cardiac arrest, *LaG* Load-and-Go, *SaT* Stay-and-Treat, *ICU* Intensive care unit, *ROSC* return of spontaneous circulation.

ROSC was achieved in 168 patients (74%) in the LaG group versus 143 patients (78%) in the SaT group (p = 0.344) (Table 2). The time to achieve ROSC did not differ between the LaG and SaT groups (15 [5, 30] min versus 10 [2, 20] min, p = 0.173). In the LaG group, 220 of 228 patients (96%) were transferred to the ICU regardless of ROSC achievement; in 44 (20% of the transferred patients), ROSC could not be achieved and the CPR was promptly terminated after ICU arrival. In the SaT group, 153 of 184 patients (83%) were transferred to the ICU; ROSC could not be achieved in 10 patients (6.5% of the transferred patients) (p = 0.001 vs. the LG group).

**Figure 2:**
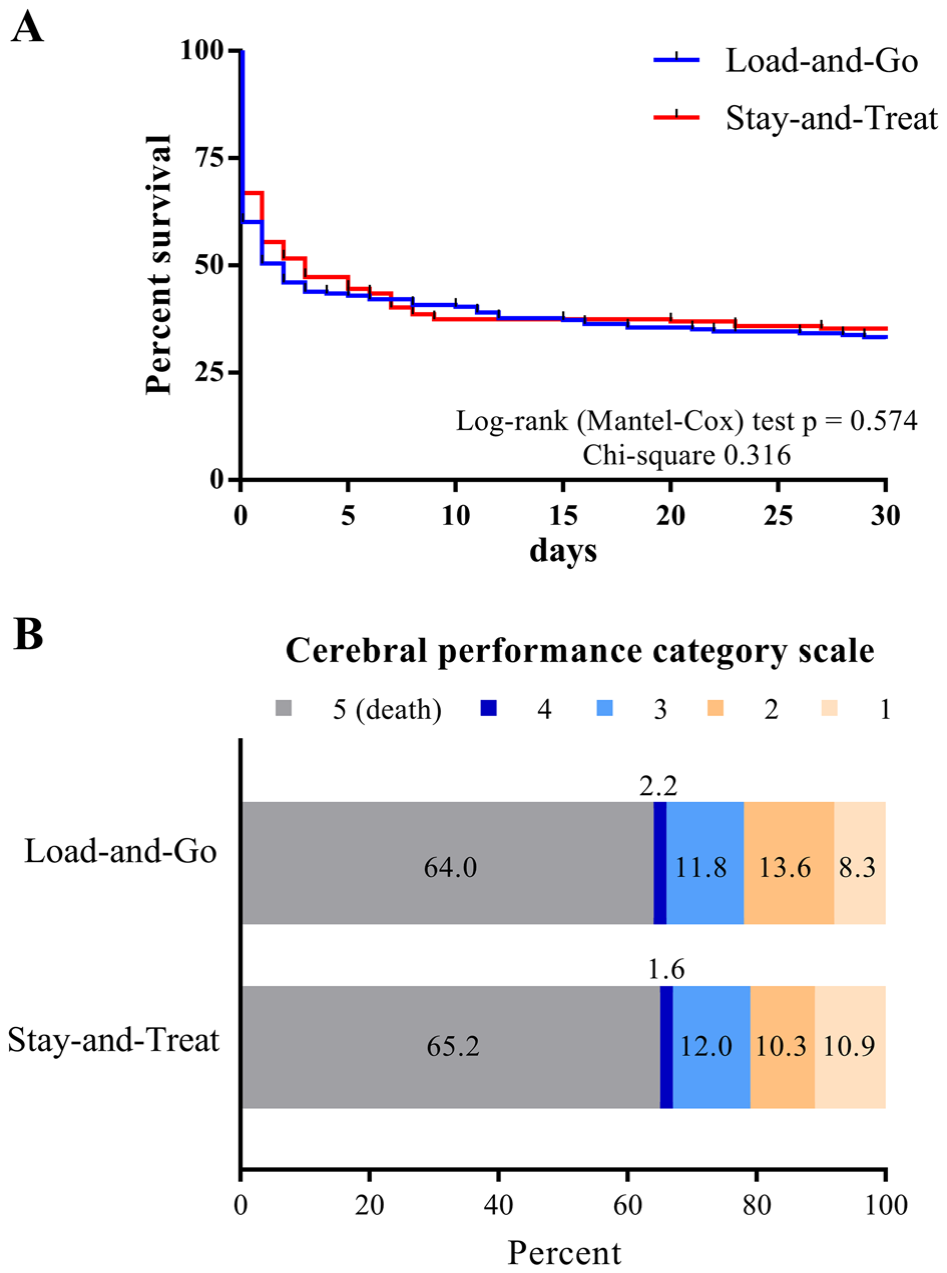
Outcome after IHCA. (**A**) Survival was similar between the two different cardiac arrest team concepts, the Load-and-Go group and the Stay-and-Treat group. (**B**) Neurological outcome was determined at the time of discharge according to the Cerebral Performance Category (CPC) scale after IHCA. CPC scale of 1 and 2 indicates good neurological outcome. Neurological outcome was similar between the two different cardiac arrest team concepts, the Load-and-Go and the Stay-and-Treat groups. *IHCA* in-hospital cardiac arrest, *CPC* cerebral performance category.

Survival to discharge did not differ between the LaG (75 patients, 32.9%) and (64 patients [34.8%]) group (p = 0.687). Kaplan–Meier curves and the log-rank (Mantel-Cox) test confirmed similar survival rates in the LaG and SaT groups (Fig. 2A).

To identify predictors of hospital mortality after IHCA, we performed a logistic regression analysis. In the multivariate analysis, a longer time to achieve ROSC, a higher first lactate level, and (in the SaT group) advanced age were independent predictors of poor survival to discharge (Tables 3, 4).

**Table 3:** LaG group: Regression analysis for hospital mortality after IHCA.

	Univariate			Multivariate		
	OR	95% CI	p-value	OR	95% CI	p-value
Age	1.026	1.002–1.050	**0.034**	1.035	0.993–1.080	**0.106**
Gender	0.786	0.457–1.351	0.383			
Diabetes	1.187	0.672–2.248	0.598			
CAD	0.890	0.514–1.539	0.676			
PAD	0.897	0.435–1.852	0.897			
GFR	0.985	0.972–0.998	0.029			
hemoglobin	0.954	0.830–1.097	0.510			
Non-cardial arrest etiology	2.991	1.706–5.244	**0.001**	0.838	0.276–2.543	0.755
Arrest time off-hours	2.438	1.355–4.385	**0.003**	2.625	0.828–8.323	0.101
Non-shockable primary rhythm	2.628	1.489–4.636	**0.001**	2.768	0.892–8.594	0.078
Time to ROSC	1.124	1.081–1.167	**0.001**	1.069	1.022–1.118	**0.004**
Lactate	1.420	1.282–1.574	**0.001**	1.347	1.176–1.543	**0.001**

Significant values are in bold.

*IHCA* in-hospital cardiac arrest, *LaG* load-and-go, *SaT* stay-and-treat, *IHCA* intra-hospital cardiac arrest, *OR* odds ratio, *CI* confidence interval, *GFR* glomerular fraction rate, *CPR* cardiopulmonary resuscitation, *CAD* coronary artery disease, *PAD* peripheral arterial disease, *ROSC* return of spontaneous circulation.

**Table 4:** SaT group: Regression analysis for hospital mortality after IHCA.

	Univariate			Multivariate		
	OR	95% CI	p-value	OR	95% CI	p-value
Age	1.031	1.006–1.057	**0.014**	1.047	**1.001–1.095**	**0.046**
Gender	1.273	0.672–2.410	0.459			
Diabetes	0.922	0.461–1.840	0.817			
CAD	0.951	0.516–1.751	0.871			
PAD	0.925	0.366–2.336	0.868			
GFR	0.981	0.963–1.000	0.052			
Hemoglobin	0.920	0.769–1.101	0.361			
Non-cardial arrest etiology	1.810	0.981–3.341	0.058	1.4887	0501–4.419	0.474
Arrest time off-hours	2.072	1.087–3.950	**0.027**	1.006	0.358–2.831	0.991
Non-shockable primary rhythm	1.889	1.004–3.552	**0.048**	1.394	0.464–4.196	0.554
Time to ROSC	1.102	1.052–1.154	**0.001**	1.118	1.039–1.204	**0.003**
Lactate	1.152	1.067–1.244	**0.001**	1.113	1.004–1.233	**0.042**

Significant values are in bold.

*IHCA* in-hospital cardiac arrest, *LaG* load-and-go, *SaT* stay-and-treat, *IHCA* intra-hospital cardiac arrest, *OR* odds ratio, *CI* confidence interval, *GFR* glomerular fraction rate, *CPR* cardiopulmonary resuscitation, *CAD* coronary artery disease, *PAD* peripheral arterial disease, *ROSC* return of spontaneous circulation.

Neurological outcomes did not differ between the LaG and SaT group. The peak NSE levels did not differ between the groups at 41 (25, 68) µg/L in the LaG group versus 37 (24, 57) µg/L in the SaT group (p = 0.315). Fifty of 228 patients (21.9%) in the LaG group versus 39 of 184 patients (21.2%) in the SaT group (p = 0.857) were discharged with good neurological outcome according to CPC scale score (Fig. 2B).

## Discussion

Here we compared for the first time two different cardiac arrest team concepts used in the management of IHCA. Switching between the two different team strategies of IHCA management at one centre made it possible to compare their survival and neurological outcomes data. We reported that the two cardiac arrest team strategies for the management of IHCA had similar survival and neurological outcomes.

The different strategies of both cardiac arrest team concepts did not impact survival (36% in the LaG versus 35% in the SaT group). In particular, the transfer of patients under ongoing CPR in the LG group; together with this finding, the delay in endotracheal intubation did not worsen the outcome. Airway management and ventilation are essential components of CPR to achieve oxygen delivery and prevent hypoxic injury. Endotracheal intubation may have adverse effects on outcomes by interrupting cardiopulmonary resuscitation and delaying timely defibrillation and epinephrine administration^16^. Bag-mask ventilation is a less complex technique than endotracheal intubation for airway management during the ACLS phase of cardiopulmonary resuscitation in patients. In a previous study, IHCA patients without preceding respiratory failure were more likely to survive IHCA at hospitals with lower rates of tracheal intubation use compared to hospitals with higher rates^17^. In contrast, in a large observational cohort study, delayed (> 15 min) endotracheal intubation during resuscitation was associated with decreased survival after IHCA^18^. In our study, intubation time was longer in the LG group, but it remained under 15 min. In both groups, adherence to guidelines was high, and the rapid initiation of CPR and early defibrillation after the recognition of IHCA were ensured. Our findings emphasise that, regardless of where the cardiac arrest occurs, early CPR initiation and early defibrillation of a shockable rhythm is more crucial for patient prognosis than ACLS measures since every minute of a delay increases the risk of patient mortality^5,19,20^.

Nevertheless, the use of a dedicated intensive care cardiac arrest team and resuscitation performance, such as the presented SaT concept might have some advantages in terms of process consistency. Team composition, team leader, and task allocation are constant irrespective of the time point and location at which IHCA occurs. The transfer of patients with ongoing CPR to an ICU can potentially hamper the efficacy of chest compressions and impair circulatory flow time, especially when the distance to the ICU is too long. In addition, an experienced team is more inclined to terminate CPR and declare death after prolonged and unsuccessful CPR without ICU transfer. In fact, in our study, CPR was terminated timely after ICU arrival without achieving ROSC in nearly 20% of the transferred patients in the LaG group than in 6.5% of the patients in the SaT group. Therefore, a dedicated ICU cardiac arrest team can take some load off the ICU as demonstrated in the present study.

Patient characteristics such as malignancy, sepsis, low functional status prior to IHCA, and renal or hepatic dysfunction have been identified as significant predictors of poor survival^3,8,9^. It was previously demonstrated that patients who are witnessed or monitored at the time of cardiac arrest demonstrate a significantly higher survival rate to hospital discharge than those who are neither monitored nor witnessed^11,21,22^. In the present study, higher patient age, longer time to achieving ROSC, and higher lactate levels were independent predictors of poor survival after IHCA. This observation is in line with those of previous studies demonstrating that patients with a non-shockable rhythm have poorer survival to hospital discharge rates than patients with a shockable rhythm. The presence of a non-shockable rhythm may reflect the delayed detection of initial shockable rhythms that progress to non-shockable rhythms. In this context, the rapid recognition of IHCA and quick initiation of resuscitation are more crucial for outcomes than details in the organisation of cardiac arrest teams.

There are several limitations to this study. The present single-centre study included patients on an internal medicine or neurology non-ICU ward; therefore, the interpretation of the results may not be generalised and transferred to other hospitals. Furthermore, the variability among countries in incidence and survival after IHCA likely reflects differences in the definitions used to identify IHCA, the proportion of cardiac arrests captured by various registries, patient populations, and country-specific factors such as do-not-resuscitate orders, and withdrawal of care^3^. Comparisons of IHCA rates between countries or registries should be performed carefully. The before-and-after study design might have been susceptible to confounding factors caused by differences in the study period. For example, the rate of IHCA occurrence decreased over the study period and was lower in the SaT group. Ultimately, we cannot explain this phenomenon. The patient comorbidity index did not differ over time. This finding may be related to the improved monitoring conditions in the normal ward at our centre. This might have facilitated the detection of physiological decline in patients earlier than later, which in some cases, the deterioration of IHCA could have been prevented. Furthermore, the study was not randomised. Although the characteristics of the patient cohorts did not differ, we only compared similar but not equally distributed cohorts.

In conclusion, here we demonstrated that both cardiac arrest team concepts for the management of IHCA did not differ in terms of survival and neurological outcomes. However, a dedicated (intensive care) cardiac arrest team can help unburden the ICU.
